# Evaluation of the mechanisms of sarcopenia in chronic inflammatory disease: protocol for a prospective cohort study

**DOI:** 10.1186/s13395-021-00282-5

**Published:** 2021-12-11

**Authors:** Amritpal Dhaliwal, Felicity R. Williams, Jonathan I. Quinlan, Sophie L. Allen, Carolyn Greig, Andrew Filer, Karim Raza, Subrata Ghosh, Gareth G. Lavery, Philip N. Newsome, Surabhi Choudhary, Leigh Breen, Matthew J. Armstrong, Ahmed M. Elsharkawy, Janet M. Lord

**Affiliations:** 1 Institute of Inflammation and Ageing, University of Birmingham, Birmingham, B15 2TT UK; 2 University of Hospitals of Birmingham NHS Trust, Birmingham, UK; 3 NIHR Biomedical Research Centre (BRC), University Hospitals Birmingham and University of Birmingham, Birmingham, UK; 4 School of Sport, Exercise and Rehabilitation Science, University of Birmingham, Birmingham, UK; 5 MRC-Versus Arthritis Centre for Musculoskeletal Research, University of Birmingham, Birmingham, UK; 6 Sandwell and West Birmingham NHS Trust, Birmingham, UK; 7 Institute of Metabolism and Systems Research, University of Birmingham, Birmingham, UK

**Keywords:** Sarcopenia, Chronic liver disease, Inflammatory bowel disease, Inflammatory arthritis

## Abstract

**Background:**

Several chronic inflammatory diseases co-exist with and accelerate sarcopenia (reduction in muscle strength, function and mass) and negatively impact on both morbidity and mortality. There is currently limited research on the extent of sarcopenia in such conditions, how to accurately assess it and whether there are generic or disease-specific mechanisms driving sarcopenia. Therefore, this study aims to identify potential mechanisms driving sarcopenia within chronic inflammatory disease via a multi-modal approach; in an attempt to help define potential interventions for future use.

**Methods:**

This prospective cohort study will consist of a multi-modal assessment of sarcopenia and its underlying mechanisms. Recruitment will target three chronic inflammatory diseases: chronic liver disease (CLD) (*n*=50), with a subset of NAFLD (*n*=20), inflammatory bowel disease (IBD) (*n*=50) and rheumatoid arthritis (RA) (*n*=50) both before and after therapeutic intervention. In addition, 20 age and sex matched healthy individuals will be recruited for comparison. Participants will undergo 4 assessment visits at weeks 0, 2, 12 and 24. Visits will consist of the following assessments: blood tests, anthropometrics, functional assessment, quadriceps muscle imaging, actigraphy, quality of life questionnaires, food diary collection and muscle biopsy of the vastus lateralis (at weeks 2 and 24 only). In addition, stool and urine samples will be collected for future microbiome and metabolomics analysis.

**Discussion:**

This is the first study to use a multi-modal assessment model to phenotype sarcopenia in these chronic inflammatory diseases. We hope to identify generic as well as disease-specific mechanisms driving sarcopenia. We appreciate that these cohorts do require separate standards of care treatments which limit comparison between groups.

**Ethics and dissemination:**

The study is approved by the Health Research Authority - West Midlands Solihull Research Ethics Service Committee Authority (REC reference: 18/WM/0167). Recruitment commenced in January 2019 and will continue until July 2021. The study was halted in March 2020 and again in January 2021 with the COVID-19 pandemic. The findings will be disseminated through peer-reviewed publications and conference presentations. All data will be stored on a secure server.

**Trial registration:**

ClinicalTrials.gov Identifier: NCT04734496

## Background

In many chronic inflammatory diseases, including rheumatoid arthritis (RA), Inflammatory Bowel Disease (IBD) and chronic liver disease (CLD) incorporating cirrhosis, loss of muscle strength, mass and physical performance (sarcopenia) is a hallmark of advanced disease, contributing to poor patient quality of life, a reduced response to challenges such as surgery or infection, and higher mortality [1]. The current working definition of sarcopenia by the European Working Group on Sarcopenia in Older People (EWGSOP) focuses on low muscle strength and either low muscle quantity or quality with poor physical performance as an indicator of severity [1, 2]. The extent of sarcopenia and the factors driving it in chronic inflammatory conditions remain poorly defined, limiting strategic or personalised therapeutic intervention.

Muscle mass is determined by the balance of protein synthesis and degradation. Anabolic signals deriving from nutrients (amino acids), physical activity and hormones such as IGF-1 and testosterone, mediate their effects through downstream targets including AKT and mTOR [3]. Catabolic signals which induce protein breakdown through induction of atrogenes such as MAFbx and MURF1, include inflammation, physical inactivity, cortisol generated in muscle via the activation of 11βHSD1 and the hormone myostatin which inhibits myoblast differentiation into mature muscle fibres and blocks AKT signalling [4–7]. The relative contribution of these mechanisms to sarcopenia in chronic inflammatory disease remains partially understood and is a major focus of this study.

### Chronic liver disease

A significant proportion of patients with CLD have a degree of malnutrition and this is closely associated with sarcopenia [8, 9]. Muscle wasting within CLD can be due to several factors including altered metabolism and inactivity. Protein-energy malnutrition is very common in patients with liver cirrhosis (estimated at 65–90%) [10]. Mechanisms reducing protein synthesis and enhancing degradation such as accelerated starvation where glycogen depletion occurs, insulin resistance and hyperammonaemia predominate in CLD [10, 11]. It is becoming increasingly clear that the degree of sarcopenia is directly related to outcomes in patients with liver disease. In a recent study of 130 patients with cirrhosis, survival at 5 years was 50% in those with sarcopenia at baseline, compared to 80% without sarcopenia at baseline [12]. In a study of 669 cirrhotic patients assessed for transplantation in Canada and the USA, sarcopenia was present in 45% of the patients and they had a median survival of 20 months compared to 95 months for those without sarcopenia [13].

In liver disease, sarcopenia may be hidden by either oedema masking muscle loss, or obesity [14, 15]. Current prognostic indicators such as the Royal Free Hospital Global Assessment (RFH GA) tool, or scoring systems such as Child-Pugh score and Model for End Stage Liver Disease (MELD) [16] score based on anthropometry/nutritional assessment of patients, may thus have reduced sensitivity in the presence of these relatively common confounders. This can underestimate the risk for those with sarcopenia and reduce the validity of assessing and prioritising patients for liver transplant surgery. In addition, since muscle mass is a static reflection of the health of the musculoskeletal system, it is also important to assess dynamic aspects such as strength, power and fatigability.

With the mounting clinical evidence of the negative impact of sarcopenia in cirrhosis, research into the underlying mechanisms is beginning to evolve. A positive anabolic effect has been observed in a small number of cirrhotic patients fed a leucine-rich preparation of amino acids [17] which suggests that cirrhotic patients should respond well to both resistance exercise and nutritional signals. Inflammation can drive muscle degradation, with inflammatory cytokines increasing activity of 11–beta hydroxysteroid dehydrogenase 1 (11βHSD1) to generate cortisol within the muscle [18] as well as promoting insulin resistance. The profound inflammatory state in cirrhosis may, therefore, be a potential driver of muscle loss.

### Inflammatory bowel disease

Sarcopenia, often included within the spectrum of malnutrition, is present in patients with IBD, including both Crohn’s disease (CD) and ulcerative colitis (UC). Malnutrition affects up to 75% and 62% of patients with CD and UC, respectively [19]. The current prevalence of sarcopenia in IBD is not fully appreciated; a recent meta-analysis found 52% of patients with CD and 37% of patients with UC have sarcopenia (as defined by low muscle mass independently) [20] with a considerable impact on quality of life.

Fatigue is a common symptom associated with IBD [21]. It is multifactorial and not necessarily associated with clinical inflammatory disease activity. Approximately 50% of patients with IBD report fatigue at the time of diagnosis [22] which can present as diminished physical activity and isokinetic muscle strength. Sarcopenia plays a role in impacting on other aspects of quality of life including a reduction in mobility, usual activities and pain/discomfort score compared with healthy controls [23]. The prevalence and clinical impact of sarcopenia in CD is poorly understood [24]. However, there are several studies that identify the negative impact of sarcopenia on post-surgical outcomes in patients with IBD, for example, increased post-operative complications. Sarcopenia can also be an independent predictor of the need for bowel resection among IBD patients [25]. There is emerging evidence of increasing obesity in patients with IBD, therefore sarcopenic obesity will likely further materialise within this cohort [20].

In relation to drivers of sarcopenia, impaired activation of muscle synthesis pathways such as the IGF1-Akt pathway may be responsible [26]. The reduction in signalling through this pathway is known to be important in the anabolic resistance of age-related sarcopenia but has not been extensively studied in IBD. A key role for inflammation as a driver of muscle wasting has been suggested by a small study which showed sarcopenia was reduced by anti-TNFα monoclonal antibody therapy [27].

### Rheumatoid arthritis

Muscle atrophy develops in two-thirds of RA patients, leading to debilitating rheumatoid cachexia which incorporates sarcopenia, shortened working life and poor quality of life [28]. As similarly reported in IBD cohorts, fatigue is a frequently reported symptom in those with RA [29]. The heightened inflammatory status of RA patients has been linked to an increase in whole-body protein breakdown [30, 31]. 11βHSD1 has been shown to be elevated in muscle from patients with RA, this may be a protective response to heightened inflammation as studies with 11βHSD1 knockout mice lacking this glucocorticoid generating enzyme developed more severe myopathy [32]. Furthermore, anti-TNFα therapy does not reverse sarcopenia, despite control of systemic inflammation, suggesting that other mechanisms are driving sarcopenia in RA [33]. The current most effective treatment to protect against sarcopenia and improve physical function in RA patients is exercise training [34]. It has been demonstrated that patients with RA are relatively sedentary with the majority of their physical activity remaining low intensity [35], however low-level physical activity can stimulate reductions in cardiovascular risk, insulin resistance and mortality [36]. In particular, a recent RCT implementing a supervised progressive resistance training in patients with RA was proven to increase muscle mass with improved function [37].

The aim of this study is therefore to determine the extent of sarcopenia in three chronic inflammatory diseases, CLD, IBD and RA, and to identify the processes, both molecular and lifestyle, contributing to sarcopenia in each disease, in order to identify common and disease-specific mechanisms. This will provide the evidence base for future studies testing interventions to reduce sarcopenia in each of these diseases.

## Methods

### Study overview

The proposed study is a single centre, longitudinal observational, prospective study of a cohort of patients with sarcopenia associated with chronic inflammatory disease, to identify generic and disease-specific mechanisms that potentially drive sarcopenia.

Patients recruited to the study will undergo several assessments (anthropometric, functional, serological, radiological, lifestyle and health-related quality of life evaluation) to assess their sarcopenia status and provide muscle biopsies on two occasions to allow further analyses including protein turnover, mitochondrial and satellite cell function, RNA sequencing, to be undertaken. Patients will be assessed at four defined time points: baseline, 2 weeks, 12 weeks and 24-week time points to allow analysis pre- and post-biologic therapy in the IBD and RA cohorts. Some of the CLD participants will undergo a liver transplant during their participation allowing a pre- and post-transplant comparison. Patients will be recruited from the University Hospitals Birmingham Foundation Trust, Birmingham, UK. Patients will continue with their standard of care management as determined by their regular specialty team. Age and sex matched healthy controls will undergo the same assessments and provide a muscle biopsy at a single study visit. They will comprise of people without sarcopenia or any significant comorbidities, with a regular level of activity (ie those without a high level of exercise/performance or not sedentary).

### Study aims

The aims of this study are:

1. To assess the extent of sarcopenia in our three target cohorts using several methods to assess muscle mass, physical performance and strength.
2. To identify potential mechanisms of sarcopenia in each disease through a multi-modal assessment process.
3. To observe any longitudinal changes in each cohort over a 6-month period.

### Study design

#### Patient selection and sample

A target of fifty patients (over the age of 18 years) will be selected from:

- Patients with CLD, predominantly liver cirrhosis or end-stage liver disease (ESLD)) who are being assessed for liver transplantation (LT). A further twenty patients with non-alcoholic related fatty liver disease (NAFLD), confirmed by transient elastography +/− liver biopsy, with significant fibrosis (Kleiner fibrosis stages F2–F4) and without ESLD will also be identified and recruited for comparison.
- Patients with active IBD who are undergoing medical management only and are due to commence a new biologic treatment.
- Patients with active rheumatoid arthritis (RA) who are commencing a new biologic treatment.

We will also recruit an age-sex matched sample of twenty healthy individuals as a control cohort for comparison.

This is an observational prospective cohort study with deep phenotyping (experimental) of each patient group to determine the mechanisms underlying sarcopenia in each cohort, therefore a sample size calculation was not applicable. However, we calculated that 50 patients per disease group is sufficient for a Spearman correlation of ≥0.3 to reach statistical significance. Recruitment targets are also based on feasibility within the time frame of the study and based on the planned statistical analysis, described below.

#### Standard of care treatment

CLD; Patients with ESLD on a transplant waiting list undergo prehabilitation (the process of enhancing functional capacity to enable the patient to withstand a forthcoming stressor [38]). This includes physical activity and nutritional optimisation [9, 39, 40].

IBD; The British Society of Gastroenterology have clear guidelines stating when treatment with a biologic therapy is indicated and are used for moderate disease +/- failure of second-line treatment in CD and UC [41].

RA: The National Institute for Health and Care Excellence (NICE) have devised a formal guidance for drug treatment in rheumatoid arthritis. This includes those with severe disease (Disease activity score (DAS) >5.1) and failure of response with combination DMARD therapy [42–44].

#### Eligibility criteria

Eligible patients will be screened via the following criteria (Table 1). CLD patients will be selected from those who undergo assessment for liver transplantation. A Non-alcoholic fatty liver disease (NAFLD) subset will be screened and recruited from the NAFLD clinic. For IBD and RA, all patients commencing a new biologic treatment (via biologic clinic screening or biologic registry screening) will be screened prior to therapy commencement.

**Table 1:** Eligibility criteria for participants with chronic liver disease, rheumatoid arthritis or inflammatory bowel disease

	Inclusion criteria	Exclusion criteria
**CLD, IBD and RA**	1. A formal confirmed diagnosis of their underlying chronic inflammatory condition: IBD cohort patients will have endoscopic or radiological evidence. Some of the CLD cohorts will have had a liver biopsy, serological and radiological confirmation will be sufficient. a. RA cohort, clinical, serological and radiological confirmation will be sufficient. b. Biologic therapy naïve on recruitment or commencing a new biologic if in the IBD or IA cohort. 2. Adults aged ≥ 18 years 3. Able to confirm written consent to the study 4. Biologic therapy naïve on recruitment or commencing a new biologic if in the IBD or RA cohort *Pre-existing or current use of immunosuppressant agents or Disease Modifying Antirheumatic Drugs (DMARDs) are acceptable in all cohorts.* 5. Meeting ACR (American College of Rheumatology)/EULAR (European League Against Rheumatism) 2010 or ACR 1987 Criteria for rheumatoid arthritis and starting DMARD therapy. 6. Meeting criteria of an inflammatory arthritis as per the American College of Rheumatology 7. Meeting criteria of liver cirrhosis including all Child Pugh scores from A-C as per British Association for the Study of the Liver guidance. 8. Meeting criteria for IBD as per the British Society of Gastroenterology guidance. 9. For muscle biopsy sampling (does not preclude patients from participating if they do not meet the below criteria) INR ≤ 1.6 Platelet count > 30	1. Refusal or lack capacity to give informed consent. 2. Currently enrolled in an interventional trial with active treatment for their chronic disease condition. 3. Previously undergone LT or biliary intervention in the CLD cohort. 4. Underlying or active cancer. 5. Biliary intervention if CLD 6. For Muscle biopsies only (able to continue in study): a. Obvious injury to both thighs. b. Active bleeding of site, pre-procedure, c. Abnormal observation parameters. d. Acute illness. e. INR > 1.6. f. Platelet count < 30. g. Anticoagulation which cannot be paused due to increased risk to pre-existing comorbidity. 7. For undergoing an MRI a. Pacemaker. b. Metal work inserted that is not MRI compatible or further information cannot be obtained.
**Healthy controls**	1. Adults aged ≥ 18 years. 2. Able to confirm written consent to the study. 3. No co-existing chronic inflammatory condition, cancer or significant premorbid disease pathology. 4. No suspicion or evidence of sarcopenia. 5. No previous transplantation.	1. Refusal or lack capacity to give informed consent. 2. Pregnancy. 3. Any recent acute illness or surgery requiring significant treatment or hospitalisation (within the last 12 weeks). 4. Any systemic corticosteroid use or replacement. 5. For muscle biopsies only: a. Obvious injury to both thighs. b. Active bleeding of site, pre-procedure. c. Abnormal observation parameters. d. Acute illness. e. INR > 1.6. f. Platelet count < 30. g. Anticoagulation which cannot be paused due to increased risk to pre-existing comorbidity.

#### Participant recruitment

Participants will be recruited from University Hospitals Birmingham, Birmingham, UK.

##### CLD cohort

All patients who attend the outpatient LT assessment clinic will be screened on the eligibility criteria. Patients with any degree of muscle wasting or functional decline will be invited to take part in the study. For the NAFLD cohort, we will screen all patients attending NAFLD clinic against the eligibility criteria.

##### IBD and RA cohorts

For RA patients, the biologics registry will be screened, and eligible participants directed towards the research team. For IBD patients, potential participants will be identified from the nurse-led biologic clinic attended by those commencing a new biologic. A selection of patients may be identified by the IBD multidisciplinary meetings during discussions of standards of care.

Invitations to participate in the study will occur via face to face interaction during clinic and patient information sheets (PIS) distribution through post or email to eligible patients by the clinical research teams. We will then contact the patient up to 4 weeks after they have received the PIS to discuss their potential participation. The patient will be free to refuse to take part or withdraw from the study at any time and this decision will not affect their care.

##### Control cohort

Poster advertising recruitment will be disseminated across the University of Birmingham, targeting staff and students. All participants who volunteer will then be screened for eligibility.

#### Study schedule and method

**Fig. 1:**
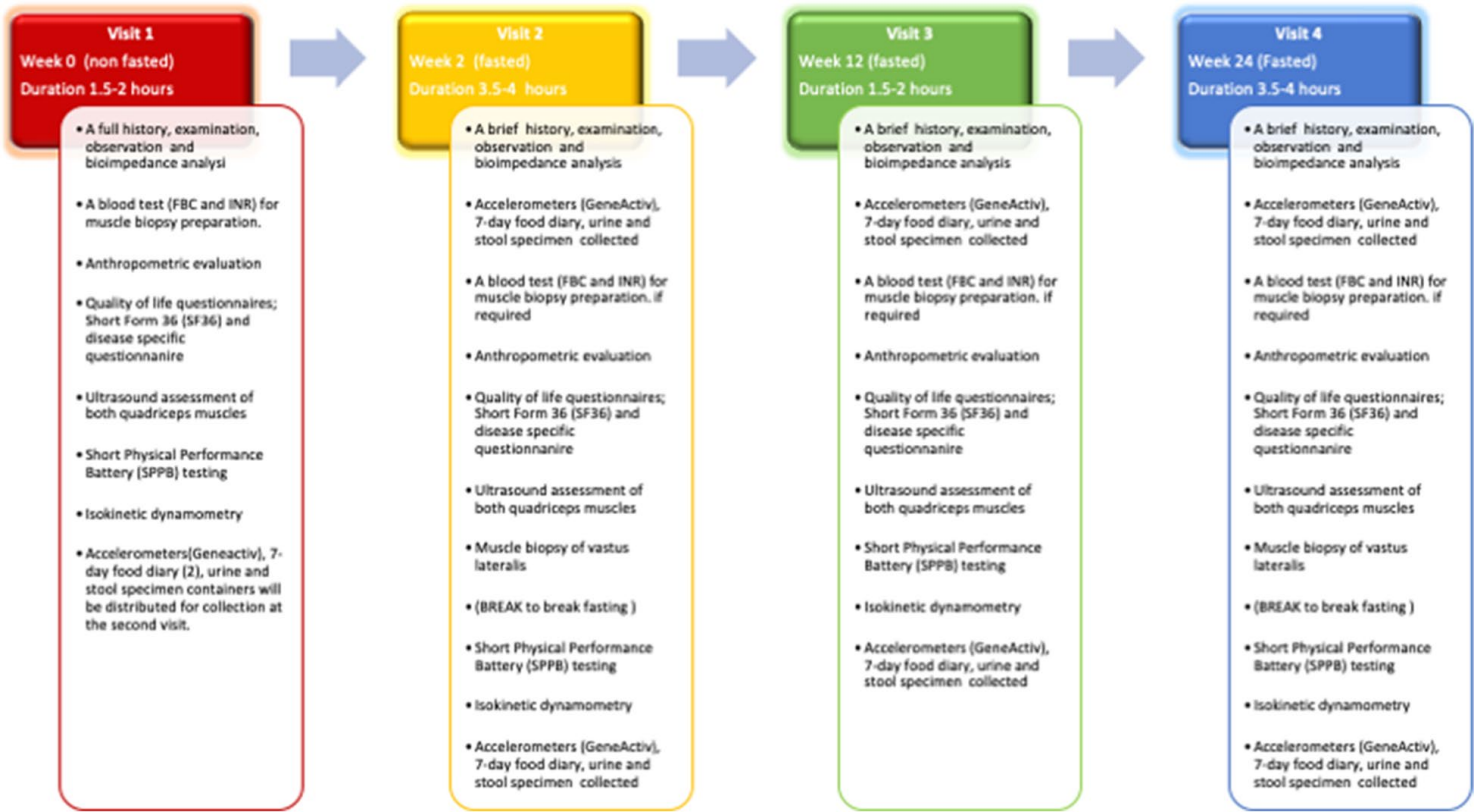
Flow chart to summarise each visit and time point of the study and the assessments performed

This study entails four visits spanning a 24-week period. Figure 1 provides an overview of the duration and assessment carried out for each visit. Some of the visits may occur outside of the selected time frames for each visit, for example, to accommodate LT, unexpected illness or events that can occur during their participation. If patients have been admitted during the timing of a visit, we will seek permission from the team overseeing their care and the patient, for participation if clinically safe and feasible to do so.

#### Experimental variables

The primary experimental variables for this study are:

1. To identify the best non-invasive assessment parameters to evaluate sarcopenia in CLD, IBD and RA and acquire data for the degree of sarcopenia in each disease cohort (muscle area, strength, functional state and activity indices).
2. To identify any longitudinal change in measures as determined by change in muscle mass, strength and function over a 6-month period, in concordance with their clinical status and treatment.

The disease-specific secondary experimental variables related to mechanisms potentially driving sarcopenia are highlighted in Table 2.

**Table 2:** Secondary outcome measures

CLD	IBD	RA
• Change in model for end-stage liver disease (MELD) scores over the 24-week period. • Complications of CLD (frequency of ascitic paracentesis, number of hospital admissions, the incidence of serious infections such a spontaneous bacterial peritonitis). • For those who undergo LT, post-operative complications, e.g. length of admission. • Transplant free survival time. • Quality of life measures. • Physical activity indices.	• Change in clinical status. • Change in mucosal healing, as defined by Travis et al, [45], symptoms scoring including Harvey Bradshaw Index scoring and severity staging including Mayo scoring. • Complications of IBD (number of hospital admissions, number of acute flares). • Quality of Life measures. • Physical activity indices.	• Change in disease activity (ACR response criteria, DAS28 response, achievement of ACR/EULAR remission criteria). • Complications of RA (number of hospital admissions, number of acute flares) • Quality of life measures. • Physical activity indices.

#### Assessment modalities

We will carry out the following assessments as described below.

- *Full clinical history and examination*: including a full set of routine observations.
- *Assessment of blood samples*: C reactive protein, ESR, baseline renal function, liver function, full blood count, clotting function, vitamin D, ferritin, folate, vitamin B12, thyroid function, lipids, and trace elements (zinc, selenium and vitamin A) [46]; cytokine and adipokine panel (interleukin (IL)IL1β, IL4, IL6, IL8, IL10, IL12, IL13, IL16, IL17, IL23, IL33, ILIRA, tumour necrosis factor alpha (TNFα), leptin, ghrelin, adiponectin) will be measured by Luminex [47]; serum cortisol, cortisone, dehydroepiandrosterone (DHEA), dehydroepiandrosterone sulphate (DHEA-S) and 3-methyl histidine (assessing skeletal muscle catabolism) by mass spectrometry [48, 49]; peripheral blood mononuclear cells (PBMCs) will be collected and stored at – 80 °C for future analysis of the degree of immune ageing.
- *Urine and stool sampling*: A 30-ml urine and 30-g stool sample from the preceding 36 h will be collected and stored for metabolomic, inflammatory and microbiome analysis (dependent on further funding).
- *Anthropometric assessment*: Age, weight, height, waist and hip circumference, and body mass index will be measured. Bioelectrical impedance analysis will be performed to estimate percentage body fat and fat-free mass [1]. Hand grip strength (HGS), mid-arm muscle circumference (MAMC) and triceps skinfold thickness (TSF) will be determined.
- *Functional assessment*: This will include accelerometry, Short Physical Performance Battery (SPPB) testing and isokinetic dynamometry.
- Accelerometry: Physical activity, sedentary time and sleep duration and quality will be evaluated using a GeneActiv accelerometer wristwatch, worn for 14 days prior to visits 2,3, and 4.
- SPPB: This measure is validated to predict mortality through the assessment of gait speed (time to walk 4 metres), standing balance and repeated chair stands (sit to stand test). This is scored out of a maximum of 12 points, with a maximum of 4 in each domain [50, 51].
- Isokinetic dynamometry: This measures strength and power via knee extension. Maximal muscle strength will be measured as the peak torque [52].
- *Muscle imaging*: Ultrasonographic (US) imaging of the quadriceps will measure muscle size, architecture and quality. Magnetic resonance imaging (MRI) will be used to assess muscle size (cross-sectional area, (ACSA), muscle volume of quadriceps and muscle quality (through intramuscular fat measures, myosteatosis and echogenicity of the muscle). All image results will undergo a review by Consultant Musculoskeletal Radiologist.
- *Muscle tissue evaluation*: A biopsy of the vastus lateralis will be performed from the dominant leg at visits 2 and 4 to evaluate muscle fibre structure and fibre phenotyping via immunohistochemistry. Biomolecular analysis of key anabolic and catabolic pathways, including genomic, epigenomic and transcriptomic analysis will be performed to give further understanding of the processes driving sarcopenia.
- An urgent blood test will be performed on the day of biopsy, to check clotting and platelet function are within criteria, if one has not already been performed within the preceding two weeks.
- *Quality of Life (QoL) assessment*: This will be measured using two validated questionnaires. All participants will be asked to complete the Short Form-36 (SF-36) health-related QoL questionnaire (Quality Metric Health Outcomes Solutions, Lincoln, USA) [53]. It is a practical, reliable, and validated measure of physical and mental health.

Each cohort will be asked to complete a disease-specific validated tool:

- CLD cohort - Chronic Liver Disease Questionnaire (CLDQ): A 29-item tool that evaluates the following domains: fatigue, activity, emotional function, abdominal symptoms, systemic symptoms, and worry [54].
- RA cohort - Functional Assessment of Chronic Illness Therapy – Fatigue (FACIT-F) Questionnaire: A 13-item tool that evaluates the following domains: physical, social, emotional and functional wellbeing to measure an individual’s level of fatigue during their usual daily activities over the past week [55].
- IBD cohort - Inflammatory Bowel Disease – Disability Index (IBD -DI): A 28-item tool that evaluates activity and participation, body structures and environmental factors [21].

*Nutritional evaluation* will be carried out for the two weeks preceding each interval review, via a 14-day food diary [8]. Diet Plan 7 (Forestfield Software Limited) will be used to calculate the macro and micronutrient intake accordingly.

Table 3 highlights the methods for performing some of the assessments in detail.

**Table 3:** Methodology for measurements

Measurement	Method
Hang grip strength	Three trials with a rest of 30 s between the tests will be performed for each hand, with a handheld Takei digital dynamometer and patients will be encouraged to exert their maximal grip strength [1, 56]. An average value will be calculated.
MAMC	This is the midpoint between the lateral edge of the acromion and olecranon process of the radius, on the mid line of the posterior surface of the dominant arm; it is measured with the arm in a supine position, flexed at the elbow with the forearm rotated against the body at 90 degrees, and the hand resting against the torso. Once the midpoint is marked, the arm returns to a supine position with the hand against the thigh. The MAMC is measured around the midpoint mark ensuring the measuring tape is even against the skin (without being taut). This will be repeated twice, and the mean result calculated.
TSF	From the midpoint calculated above, a vertical pinch with a Harpenden calliper, parallel to the long axis of the arm, is made at the landmark in a perpendicular direction. Measurement in millimetres will be taken; it will be repeated twice, and a mean is calculated [57–59].
Isokinetic dynamometry	This measures strength and power via knee extension. Maximal muscle strength will be measured as the peak torque [52]. Patients will be seated in an adjustable, straight-backed chair with their hips and their knees flexed at 90°; their pelvis and the thighs will be secured by a broad strap with their arms crossed in front of their chest. After the instruction and 2-3 practical trials, the patient will be asked to extend, then flex the leg with maximal effort, for 5 repetitions. Both legs will be tested, unless contraindicated by pain or other symptoms. Unilateral limb tests will be performed on the non-biopsied leg, following a muscle biopsy [60, 61].
US scan	Patients will lie in a semi-supine position with legs resting flat on a bed. All images will be taken at 50 % of femur length (measured from the greater trochanter to the lateral knee joint space). The maximal anatomical ACSA of human quadriceps is shown to be at ~50% of the femur length [62]. Two-dimensional B-mode ultrasonography Esoate MyLab Alpha point of care ultrasound, 4.6 cm probe (SL1543, 13-4Mhz scanning frequency)) will be performed. Three longitudinal scans will be taken at 50% femur length of the vastus lateralis muscle with the probe aligned to the fascicles; allowing for quantification of fascicle length and pennation angle. Vastus lateralis muscle thickness (defined as the perpendicular distance between the superficial and deeper aponeurosis) will be obtained. All variables will be obtained offline via image J imaging software and will be presented as a mean. For assessment of all quadricep muscles, two extended field of view ultrasound images will be taken at 50% femur length; this will allow for the quantification of quadriceps ACSA. Echogenicity can be determined using a computer-assisted grey-scale analysis offered by ImageJ [63, 64].
MRI femur	Axial and sagittal plane scans of each thigh will be obtained using an MR 3T scanner for higher quality, efficient imaging capture and 3D reconstruction. A T1 weighted Spin Echo protocol will be used (repetition time 600ms, echo time 15.2 ms, Field of view 512 × 512 mm, slice thickness 10mm, no gap between slices). Patients will lie supine on a preparation bed for up to 20 min to allow fluid shift stabilisation. A series of axial plane scans along the entire length of the quadriceps muscle group and sections at the L3 lumbar spine (L3 lumbar spine to quadriceps insertion on the tibia) will be collected. The contours of the quadriceps will be digitalised offline using the Osirix DICOM image analysis software (Pixmeo, Geneva, Switzerland) and the quadriceps muscle volume will be calculated [65]. 3-dimensional acquisition of both thighs and a Dixon sequence will be performed for fat analysis. Quadriceps muscle ACSA will be measured as described in the above. This midpoint of each femur length and the midpoint of VL (which is where ultrasound CSA measurements are taken from) will be marked with an external marker to ensure this is identified on MR images for analysis purposes and comparisons with ultrasound measurements. All image results will undergo a review by Consultant Musculoskeletal Radiologist.
Muscle biopsy	The patient will be fasted for ~6 h prior to the procedure. The ultrasound performed will identify the correct position to ensuring the sample is taken from the muscle belly. The non-dominant limb will be used if the dominant limb is not feasible. A small area of skin overlying the outer thigh will be cleaned with iodine solution. 5–10ml of 1% lignocaine is infiltrated into the subcutaneous adipose tissue and down to the muscle. After adequate anaesthetic, a small incision is made in the skin (approximately 5–7mm in length). A needle is inserted into the muscle and a small amount of muscle is taken using a well-described technique with a Bergstrom needle. A few passes may be performed. The incision will be closed using ‘steri-strips’ and a single suture, if required. A small dressing will then be placed over the biopsy site. Pressure and an ice compress will be applied to the area for 10 min by hand. A pressure bandage which will stay on for 8 h to decrease the risk of bruise formation. Patients will be asked to keep this area dry for at 3–5 days. All patients will receive after care advice and contact if any problems do arise.

### Statistical analysis plan

All quantitative data will be entered into a purpose-designed database and exported for statistical analysis in relevant statistical software. Members of the research team, including a statistician, will conduct the analysis. The analyses that will be performed will include:

1. The extent of sarcopenia in the three target chronic inflammatory diseases using several methods to assess muscle mass, function and strength and compare these data with the EWGSOP criteria.
2. Identification of potential mechanisms of sarcopenia that occur in CLD, IBD and RA achieved through a multi-modal assessment process.
3. Assessment of any longitudinal changes in each cohort over a 6-month period following their standard of care.
4. Assessment of the best non-invasive assessment parameters for evaluation of sarcopenia will be made via analysis of the results of anthropometric, functional, and imaging data, using non-parametric analysis via Spearman correlation. Specifically, we will carry out the following:
  Baseline descriptions of clinical parameters and sarcopenia with proportions demonstrated and median and mean formulated.
  Correlation analysis of measures of sarcopenia and how they relate to disease severity, QoL, etc., will be performed by qualitative measures.
  Comparisons between disease types will be made using non-paired, non-parametric testing.
  Dynamic changes over 6-months (pre and post 6-months of standard of care treatment) will be assessed using paired, parametric and non-parametric testing.

### Adverse event management

An adverse event is defined as any untoward medical occurrence in a subject and does not necessarily have a causal relationship with this study. Potential adverse events would include any side effect or complication post invasive assessment including serological testing and muscle biopsies. Patients will be consented for each individual procedure performed.

Adverse events will be graded according to the Common Terminology Criteria for Adverse Events (CTCAE), the Principal Investigator will be informed immediately, and the appropriate action will be taken, with the event recorded in the CRF.

A serious adverse event (SAE) is an adverse event that fulfils one or more of the following criteria: results in death, is immediately life-threatening, requires prolongation of existing hospitalisation, results in persistent or significant disability or incapacity, or is an important medical condition. The causality of a serious adverse event will be assessed by the investigators and recorded on the SAE form. All SAEs will be notified to the sponsor’s Research and Development department via the SAE form in the case report form (CRF). Only those events classified as probable or related will be reported to the Research Ethics committee.

### Data management

All data for an individual participant will be collected by the PI or their delegated nominees and recorded in the case report form (CRF). Participant identification in the CRF will be through their unique Participant Study Number allocated at the time of consenting for the study. Data will be collected from the time the patient is considered for entry into the study through to completion of the study. All clinical data will be stored as per NHS regulations and held on a protected database. We will require some data which may be patient identifiable from Hospital Episode Statistics; however, these data will then be inputted in a non-identifiable manner under the standardised data collection guidance to ensure confidentiality and security.

Data from the CRF will be entered onto a secure password protected RedCap database held on a password-protected area within a University Hospitals Birmingham (UHB) NHS Foundation Trust server. Some research data may also be transferred to the University of Birmingham anonymously for further research purposes. Patients will be consented for this. Due care will be taken to ensure data safety and integrity, and compliance with the Data Protection Act 2018. All essential documentation and trial records will be stored in conformance with the applicable regulatory requirements and access to stored information will be restricted to authorised personnel. Coded research data will be stored for 10 years anonymously under the property of UHB NHS Foundation Trust in keeping with good clinical practice.

### Case report forms

CRFs will include baseline/follow-up medical history and physical examinations, comorbidities and concomitant medications. Additional information incorporated in the electronic database will include basic clotting and full blood count, observations, anthropometric data, bioimpedance analysis, completion of ultrasound, MRI, and muscle biopsy (visit 2 and 4 only), recorded for each visit. Other CRFs will include AE reporting.

### Patient and public involvement (PPI)

We designed the study with ongoing participation of specific patient groups. We held an initial forum where we presented the study concept and design to a PPI group and received feedback which was incorporated into our design. All patient information sheets and documentation were reviewed by the Liver and Gastroenterology PPI group and a Rheumatology PPI group (at UHB) independently and modified accordingly. During the study, we have had ongoing input from the participants, some of which has been implemented during their experience.

A summary of the results will be disseminated to all participants at the end of the study following analyses.

### Monitoring and quality assurance

The study will be conducted according to the current guidelines for Good Clinical Practice (GCP). Any deviation, that does not result in harm to the trial subject or significantly affect the scientific value of the study, will be documented in the CRF and appropriate corrective or prescriptive actions taken.

Any serious breaches will be immediately reported to the Principal Investigator who will take whatever immediate action is required to safeguard the wellbeing of the participant. The Chief Investigator will notify the Sponsor immediately and the Ethics committee within 7 days of becoming aware of the serious breach.

### Dissemination and publication

Results will be presented at relevant national and international conferences and submitted for publication in peer-reviewed open access journals. Participants will be asked if they would like to be informed of the study results at the time of obtaining consent.

### Research governance

The study will be carried out in accordance with the Declaration of Helsinki, under Good Clinical Practice guidelines. This study is not a Clinical Study of an Investigational Medicinal Product, and thus is not governed by the Medicines for Human Use (Clinical Trials) Regulations 2004.

## Discussion

This is the first observational study to deep phenotype sarcopenia using a multi-modal assessment method of both mass, function and strength and quality of life measures.

### Justification of study cohorts

Chronic inflammation is believed to result in an environment which contributes to sarcopenia, though the exact mechanisms remain unclear and may vary between different diseases. We selected this spectrum of chronic inflammatory diseases as in their extreme, acute presentations, the degree of sarcopenia can be profound. We recognise that the standard of care does vary within these cohorts, with biologic treatment in the RA and IBD cohorts and prehabilitation in the CLD cohort). However these were chosen to represent the spectrum of chronic inflammation. This will allow us to observe the mechanisms and findings across this wide spectrum. The CLD cohort comprise of patients with decompensated liver disease, who are in the end stage of their chronic inflammatory insult. In addition, the NAFLD subset represents early stages in a chronic inflammatory process with some of these patients having potential reversible fibrosis and in comparison, to the patients with ESLD, these patients have compensated liver disease. The RA and IBD cohorts will reflect those with a significant degree of disease severity to require treatment with a biologic agent. Whilst we appreciate that we can compare only some of the elements between cohorts, it will highlight possible trajectories of sarcopenia with treatments which may be further investigated.

### Challenges in study design

We have tried to incorporate previously validated measures of sarcopenia to compare to newer evolving assessments. For example, the using of validated L3 lumbar muscle cross-sectional area [50, 66–68], HGS of non-dominant limb, SPBB as a measure of functional capacity [69], and comparing these to more novel techniques such as ultrasound analysis of quadriceps muscles [70–72], and functional variables such as isokinetic dynamometry and physical activity [73, 74]. Importantly, segmental sarcopenia can occur in patients, therefore adequate assessment of global function is imperative to determine their degree of sarcopenia, and subsequent treatment and mortality risk especially in those undergoing liver transplantation assessment [66, 68, 75].

We are also investigating factors that affect sarcopenia such as sleep efficiency and quality [76, 77] and hope to correlate this to mass and functional variables. These may be important factors that should be considered to ameliorate sarcopenia.

Covert hepatic encephalopathy is frequent in patients with end-stage liver disease and as a result can affect cognition and is of relevance to this study, namely the ability to consent [78]. Many patients are commenced on rifaximin to treat and manage this. We will tackle this by ensuring an adequate period for the patient to read the PIS, having a family member or friend present during visits, reconfirming consent at each visit and performing full capacity testing on initial review and at each required interval. We will exclude any patients who had severe HE which would impact their ability to consent and consistently and adequately follow instruction and most importantly for their own safety.

### Confounding medications

Most patients with inflammatory disease are treated with a variety of medications. For the purposes of this study, we have allowed those on oral steroids or DMARDs in all cohorts. Additionally, those within the CLD cohort may have their follow up visits post liver transplantation, for which they will be treated with immunosuppression. Given it is unsafe to pause any of these, and this is an observational study, we are recording these data. We recognise it may compound muscle loss.

### Muscle biopsy

This is the first study to attempt large samples of sequential muscle biopsies in these cohorts. It will allow the collection of safety data, however importantly, it allows comparison and analysis of functional and mass measures with muscle histology.

### Safety

Whilst the main stay of this study is observational, there have only been a few studies which have performed muscle biopsies of the vastus lateralis in patients with significant comorbidities such as liver cirrhosis [79]. We will use a Bergstrom needle which is safe, uses a small incision and therefore has a reduced risk of infection and bleeding. Also, using the vastus lateralis is safer as there are limited motor units, with no major blood vessels or nerves traversing the biopsy site [80]. We will perform an ultrasound of the thigh prior to biopsy as part of the study; however, we will use this to additionally check for the optimum site, the presence of any aberrant vessels, and the depth from skin to muscle, to aid the biopsy procedure. A stringent safety checklist is in place to ensure that every patient is adequately risk assessed including allergies, equipment and personnel present. This includes a pre-biopsy check of their INR and clotting. A cut off of INR ≤ 1.6 and platelet count ≥30 will be used in concordance with national and local radiology guidance for procedures entailing a similar risk profile [81, 82]. All medications will be checked and any antiplatelet or anticoagulation medications will be noted. A biopsy procedure will not be undertaken in those for whom stopping medication may incur significant risk, e.g. warfarin for atrial fibrillation and increased stroke risk. If feasible, medication will be stopped for a minimum of 3 days in advance, in accord with drug half-life and national guidelines for endoscopic procedures [83].

All patients will have a point of care blood sugar (BM) checked pre biopsy procedure as those with diabetes and liver disease are at risk of hypoglycaemia from fasting. Those on any medications that need to be paused or hypoglycaemic including insulin will be contacted in advance and advised accordingly on adjustment of insulin doses the day prior to and of the biopsy and visit [84].

### Future consideration

At the end of the study, each participant will be invited to attend a focus group to

feedback on their experience of the study. This study will provide baseline data for consideration of a follow-on study focusing on intervention to potentially reverse or reduce the progression of sarcopenia in this patient group.

## Conclusion

This is the first study to use a multi-modal assessment model to phenotype sarcopenia in a spectrum of patients with chronic inflammatory disease. By selecting these diverse cohorts we hope to identify the common mechanisms driving sarcopenia whilst appreciating the individual differences that may arise. This unique comparison may highlight differences that need to be considered when assessing and treating sarcopenia across the spectrum of chronic inflammatory disease. We appreciate that these cohorts do require a separate standard of care treatments which limit comparison between groups. The enrolment of participants to the study commenced in January 2019 and is due to complete in July 2021. The full analysis is expected by Q1 2022.

## Data Availability

Not applicable.

## Abbreviations

RA: Rheumatoid arthritis
IBD: Inflammatory bowel disease
CLD: Chronic liver disease
EWGSOP: European Working Group on Sarcopenia in Older People
RFH GA: Royal Free Hospital Global Assessment
MELD: Model for End Stage Liver Disease
UKELD: United Kingdom Model for End Stage Liver Disease
11βHSD1: 11–beta hydroxysteroid dehydrogenase 1
CD: Crohn’s disease
UC: Ulcerative colitis
HMB: β-Hydroxy-β-methylbutyrate
PUFA: Polyunsaturated fatty acids
ESLD: End-stage liver disease
LT: Liver transplantation
IA: Inflammatory arthritis
EULAR: European League Against Rheumatism
NAFLD: Non-alcoholic fatty liver disease
IL: Interleukin
DHEA: Dehydroepiandosterone
DHEA-S: Dehydroepiandosterone sulphate
PBMCs: Peripheral blood mononuclear cells
HGS: Hand grip strength
MAMC: Mid-arm muscle circumference
TSF: Triceps skinfold thickness
SPPB: Short Physical Performance Battery
MRI: Magnetic resonance imaging
ACSA: Cross-sectional area
DEXA: Dual-energy X-ray absorptiometry
CT: Computerised tomography
SF-36: Short form-36
CLDQ: Chronic liver disease questionnaire
FACIT-F: Functional assessment of chronic illness therapy – fatigue
IBD –DI: Inflammatory bowel disease - disability index
PIS: Patient information sheet
CTCAE: Common terminology criteria for adverse events
SAE: Serious adverse event
CRF: Case report form
UHB: University Hospitals Birmingham
